# Market violence through destructive entrepreneurship: Assessing institutional responses to the proliferation of counterfeit traditional and alternative medicines in Ghana

**DOI:** 10.1016/j.heliyon.2023.e13881

**Published:** 2023-02-21

**Authors:** Frederick Ahen, Kwame O. Buabeng, Outi M.H. Salo-Ahen

**Affiliations:** a Turku School of Economics, University of Turku, Turku, Finland; b Kwame Nkrumah University of Science and Technology, Faculty of Pharmacy and Pharmaceutical Sciences, Department of Pharmacy Practice, College of Health Sciences, Kumasi, Ghana; c Åbo Akademi University, Faculty of Science and Engineering, Pharmaceutical Sciences Laboratory (Pharmacy) and Structural Bioinformatics Laboratory (Biochemistry), Turku, Finland

**Keywords:** Alternative medicine, Consumer protection, Counterfeit medicines, Destructive entrepreneurship, Emerging markets, Herbal medicines, Institutional responses, Market violence, Patient safety, Public health

## Abstract

**Purpose:**

This multidisciplinary study seeks to determine the nature and structure of the informal markets for counterfeit medicines, the co-factors underpinning the demand and supply of counterfeit Western allopathic medicines (WAM), traditional and alternative medicines (TAM), and potential institutional responses in Ghana.

**Method:**

This study is based on an interpretive research approach. It deploys a synthesis of a longitudinal ethnographic fieldwork, with multiple repeated visits for observations, analysis of documents, interviews, and focus group discussions.

**Findings:**

The study identifies five major inter-related discoveries that point to the need for urgent institutional responses: Approaches to global health governance pay little attention to the complex economic gamut of TAM, including herbal medicines. The rise in necessity entrepreneurship and the availability of easy-to-use packaging and advertising technologies have made TAM a major competitor of WAM. The informal markets for WAM and TAM are structured in ways that allow them to evade formalized interventions and regulations. Standardization allows destructive entrepreneurs to derive advantage from economies of scale and reduce production costs, allowing the sector to flourish with little economic risk while inflicting violence on consumers. Personalization and co-creation of medicine with consumers has the added psychological effect of increasing consumer confidence. This, however, enlists consumers in the market violence against themselves.

**Social implications:**

Destructive entrepreneurship, whether inadvertent or criminal creates benefits for groups and individuals but negatively affects public health on various levels.

**Originality:**

Mitigation and interventions that ignore the informal TAM market of destructive entrepreneurship only answer a part of the big question of how to guarantee patient/consumer safety from the threats of all counterfeits.

## 1 Introduction

According to the World Health Organization's ‘Traditional Medicine Strategy: 2014–2023’ [1], traditional medicine is “*the sum total of the knowledge, skill, and practices based on the theories, beliefs, and experiences indigenous to different cultures, whether explicable or not, used in the maintenance of health as well as in the prevention, diagnosis, improvement or treatment of physical and mental illnesses*”. The WHO also reports that globally, a significant number of people seek traditional and alternative medicines (TAM) compared to conventional Western allopathic medicines (WAM) [2]. This is due their affordability, accessibility, and availability besides their sociocultural and spiritual significance. TAM such as herbal medicines play a significant role in the public health architecture of emerging economies. Close to 80% of Africa's 1.4 billion inhabitants make use of TAM [3]. While the figure for fake TAM may not be known (albeit a growing phenomenon), the WHO has reported that Africa alone accounts for 42% of all global cases of counterfeit and substandard medicines [4]. This means that 1 in 10 of all medicines on the market in Africa is possibly falsified. Within the context of the issue at hand, the proliferation of unregistered and fake/counterfeit TAM and WAM has become a serious public health concern [[5], [6], [7]]. For example, in Ghana, our research context, “antimalarials, analgesics, antibiotics, anthelmintics, and uterotonics” are highlighted as being among the most common fake medicines on the market [8]. The above global and regional statistics provide answers to the question regarding the extent to which the proliferation of counterfeits affects consumers/patients globally and resource-constrained settings in particular. Such destructive activities on the market have vast implications for national and global healthcare delivery [5].

Ground-breaking advances and significant leaps in pharmaceutical and medical technologies have brought much brighter hope to global health [9]. At the same time, such advances have increased patients’ expectations [5,[10], [11], [12]]. That notwithstanding, the impact of these improvements has not always been felt by some populations in emerging economies due to a lack of access to quality healthcare and pharmaceuticals owing to the overall weakness of the public health system [13,14]. More precisely, emerging economies pay 20 times more for life-saving medicines than industrialised economies [15], provided such medicines are available in the first place. Fake WAM (copies of generic and branded medicines produced by the pharmaceutical industry) and TAM (especially herbal medicines) without registration or marketing authorization keep flourishing on the market to fill the void [5,7,10,[16], [17], [18], [19], [20]].

Although the informal markets (of medicines) in emerging economies represent the least understood locus of competition for both quality and spurious medicines, the socio-psychological and public health aspects of the demand and supply of medicines are often ignored in most analyses. This gap stems from the fact that local TAM are erroneously perceived as non-technical and non-scientific. Besides, health, in general, is ill-defined in many ways as it precludes community-level implications [21]. Pharmacists, however, do not take the major role of TAM lightly. This is because recent toxicological analysis and surveillance reports indicate that with long-term exposure, such medicines pose a potential danger to patients on several orders of magnitude; they can cause cancer and organ health risks to consumers [5,7,22]. More prominently, the proliferation of counterfeit medicines in emerging economies poses massive threats to public health and socio-economic growth. Selling counterfeit medicines is a form of market violence (i.e., the harm or suffering inflicted by the logic of the market [23]) that affects millions of people to various degrees. Nevertheless, the focus of global interventions primarily on WAM has meant that less attention is paid to the underregulated and mostly informal TAM industry (the manufacturing conditions, commercialization, safety, and efficacy of products), although it enjoys a significant market share.

It is instructive to note that since the outbreak of the coronavirus disease (COVID-19 pandemic), there have been several reports of unregistered and counterfeit TAM being used to treat the disease globally [[24], [25], [26], [27]]. Regrettably, there is a market for such goods because of a lack of access to regulated and controlled medicines. The diffusion of self-medication among the public also plays a major role in people's patronage of non-evidence-based medicines [28]. In many cases, distrust for institutional recommendations for therapeutic and prophylactic interventions [29,30] has fed the need for self-medication. Unfortunately, in many cases, this has also led to the development of antimicrobial resistance [31].

In this contribution, we analyse and synthesize data from longitudinal fieldwork to determine the co-factors that influence consumer preferences for counterfeit WAM and unregistered/counterfeit TAM [18,19,32] in their diverse forms and the institutional responses to that. We use Ghana as a proxy for the emerging markets of West Africa given the countries’ similar public health profiles and uniform coordination in combatting counterfeit medicines. By institutional responses, we are referring to regulatory, educational, advocacy, policy, R&D, and medico-technoscientific interventions aimed at maintaining the integrity of the value chain of WAM and TAM to ensure patient safety. More prominently, we answer the question:

How do public health actors (managers/entrepreneurs, policymakers, and clinicians) create the maximum social value with and for consumers/patients through policy innovations (institutional responses) against counterfeits/unsafe medicines?

Here, ‘creating maximum social value’ is conceptualized as the socioeconomic, political, and public health processes of guaranteeing the highest-quality medicines and associated services that deliver optimal health benefits to patients whilst reducing potential harm. This is seen as the result of a wholly protected value chain whose integrity is uncompromised to ensure consumer/patient safety whilst increasing the trust in public health systems. Since its formation in 1948, the WHO has defined health as “*a state of complete physical, mental, and social well-being, and not merely the absence of disease or infirmity*” [14]. This definition leaves out the fundamental pillars of health in terms of community and population health where inequality and income disparity or forms of discrimination affect health outcomes [13]. This is because health is much more than the absence of disease suffered by individuals [13,21]. Therefore, the question being posed covers the multidimensional aspects beyond medicalized conceptualizations to unpack the social value that is destroyed with counterfeits or created with institutional interventions. For example, a breadwinner who loses money through the purchase of counterfeits and endangers his/her health does not only affect the individual but whole households and even communities. The widespread effects of such incidents have ramifications on national socio-economic development and general well-being. Our main thesis in this study is that global or public health interventions, policies, and medico-technoscientific structures that do not simultaneously prioritize patient protection from fake WAM, and unregistered/spurious TAM undermine sustainable healthcare delivery in emerging economies in Africa where the informal sector is a significant provider of care.

## 2 Theoretical positioning: destructive entrepreneurship

Entrepreneurship is uncritically associated with innovation, wealth creation, and an increase in general welfare through economic activities. Baumol's [33] pioneering work and several others suggest otherwise. Shepherd [34] describes “*the destructive side of entrepreneurship as the negative impacts on society members from damage to resources owned or accessed by others as a result of entrepreneurial action*”. From a jurisprudential perspective, destructive entrepreneurship is conceptualized as any illegal entrepreneurial activity that is linked with organized crime and/or economic crime [35,36]. For our purpose, this definition does justice to counterfeit production and sales.

Lucas and Fuller [37] expand on the works of Baumol [33] by arguing that there is an inherent ambiguity between institutions and entrepreneurship. Although social value creation is the outcome of entrepreneurial activities in supportive institutional contexts, entrepreneurs by nature may either create or destroy value in any institutional environment. They raise the old question about the institutional conditions under which entrepreneurs have the propensity to create the maximum social value. They argue that “social value creation depends on the entrepreneur's next best alternative, and institutions are constraints on the relevant alternatives”. Thus, destructive entrepreneurship refers to an economic activity in which actors expend resources on rent-capturing or wealth expropriation [37].

In the ideal textbook world, pharmaceutical markets are natural responses to consumer/patient needs and wants. However, compelling evidence suggests that markets are first the consequences of entrepreneurs' quest to maximize utility and then hopefully that of the consumer. In fact, Adam Smith [38] established this economic notion centuries ago. Indeed, the fundamental reason for the existence of most fake medicine businesses is a hundred percent profit-oriented with zero regard for patient safety. Entrepreneurs are opportunity-seeking and risk-taking by nature. They seek to maximize their utility (rent-seeking) ‘wealth, power and prestige’ [39] by creating new value, product, or service that fulfils a need on the market. Under supportive institutional conditions, such activities must be legal and ethically acceptable. However, when such ventures are operated in ways that deviate from legal, moral, and institutionally acceptable modus operandi, they do not always lead to productive outcomes for society and stakeholders or consumers [35].

There are many nomenclatures given to activities that produce negative outcomes. This is because they deviate from statutory and mandatory legal and ethical principles. In his seminal work, Baumol [33,39] clarifies the differences between three categories of entrepreneurship: productive (value-creating, new wealth, innovative processes and products), unproductive (non-value adding), and destructive (value destroying). Within the context of counterfeit medicines, the two latter categories not only fail to create new value but destroy value due to their fraudulent and criminal nature (adversely affecting human health, siphoning customers from legitimate firms, and evading taxes). To Sautet [40], there is productive, evasive, and socially destructive entrepreneurship. In evasive entrepreneurship or criminal networks [41], actors find ways to elude formal institutions or rules of the game. Henrekson [42] talks about predatory entrepreneurship (when economic agents take advantage of or abuse the system for personal gains). Tierney and Tepper [43] simply refer to such behaviours as destructive leadership. More prominently, destructive entrepreneurship takes place in the activities that exploit natural resources for purely rent-seeking purposes without sustainable strategies for their renewal [44]. Within the current context, we can refer to unlicensed open street/internet vendors/retailers or entrepreneurs as people operating through market violence [23]. For some operators, their economic conditions force them to sell whatever people buy albeit legally unjustifiable and potentially dangerous to patients whilst for others, there is a criminal intent behind their behaviour given the non-stringent regulatory controls.

Most studies in international business journals focus on the operations of tax-paying, employment-creating, and innovative small and medium-sized enterprises (SMEs) or multinational companies (MNCs), or start-up firms that promote economic growth [45], or firms that are just unproductive. A neglected conceptual gap however is the activities of destructive forms of entrepreneurship [33,34,37,44]. Typical examples are the mafia and other criminal organisations (e.g., cross-border contraband manufacturers and distributors) and the extent to which they impose costs on the legal economy [41,46]. Most crime-focused studies in economics and business largely deal with criminal activities that lead to destructive outcomes. Here, for our purpose, however, fake WAM that are packaged as authentic, legal, and approved for consumption infiltrate even legitimate hospitals, pharmacies, and chemical shops to confuse both clinicians and consumers—causing extended damage. Institutional environments determine the formal and informal rules of the game and the allocation of incentive structures [47] for the flourishing and proliferation of counterfeit-producing enterprises. In essence, at the micro-level, destructive entrepreneurship benefits individual risk-takers while negatively affecting the macro level or economic growth by lowering the GDP [44]. Table 1 below shows the magnitude of counterfeit medicines at the top of the pack of illegal forms of entrepreneurship.

**Table 1:** Top illicit goods ranked by financial value^a^.

Illegal market product or activity	Estimated value in billions of USD
Counterfeit pharmaceutical drugs	200
Prostitution	186
Counterfeit electronics	169
Marijuana	142
Illegal gambling	140
Cocaine	85
Prescription drug abuse	73
Heroin	68
Software piracy	63
Counterfeit foods	49
Methamphetamine	28
Ecstasy	16
Counterfeit pesticides	6
Arms trafficking	1

a Source: Havoscope.com (2019).

It is interesting that most studies hardly mention the absence of ethical foundations in the entrepreneurial activities that negatively affect consumer health. This is where destructive entrepreneurship ties with market violence as a theoretical addition.

### 2.1 How does destructive entrepreneurship tie with market violence?

The term market violence refers to the harm or suffering inflicted by the logic of the market in both symbolic and material terms [23]. This happens especially when consumers (and patients for our purpose) are extremely vulnerable [48,49], thereby paving the way for exploitation through deceit and fake products. While such situations are bad enough in sectors such as short-term lending and banking at high-interest rates [49,50], deceptive and predatory advertising leads to addictive consumption of health-ruining products [51]. It is worse when it comes to the area of direct and active consumption of pharmaceuticals for important health needs [52]. Such products inflict not only economic harm but psychological and other health-related effects on consumers' overall health even with long-run consequences. A fake or a knock-off Armani, Versace, or Nike product can still be worn with affordable regrets of having been ripped off. However, they are certainly not health-ruining like counterfeit medicines that are consumed to affect a person's biological makeup and well-being. The level of harm caused by counterfeit medicines is disturbing on various levels, hence the need to address the co-factors that influence consumer preferences for counterfeit WAM and TAM [18,19,32].

Market and governance failures are partly responsible for the counterfeit crisis because of the void they create by not attending to the low-income populations [53]. Such failures were highly pronounced during COVID-19 with vaccine nationalism and vaccine apartheid [54,55]. Additionally, the outbreak of COVID-19 has exacerbated the proliferation of counterfeit cures that promise all kinds of wonderful solutions in the form of prophylactics and therapeutics. Destructive entrepreneurs also go beyond cures to provide even forged or fake COVID-19 certifications.

Additionally, the articulation of cure, based on WAM, is also dominated by international private and supranational organisations (e.g., WHO) and has led to apathy towards traditional medicine [12], thus leading to limited R&D and innovations related to new herbal cures [9]. Herbal medicines are generally referred to as traditional or alternative medicines. These terms are often used interchangeably. Despite their popularity and usefulness [6,56], TAM are sometimes pejoratively referred to as ‘unproven’ or lacking ‘enough evidence’. The focus of interventions is more on WAM and therapeutic measures, which, although necessary, must be seen as a last resort. Instead, health and prevention must be the focus. The next section outlines the methodological approaches and research setting of the study.

## 3 Methods

### 3.1 Research setting and context

Before zeroing in on the specific localities that were studied, this section offers a brief description of Ghana's health sector in terms of overall governance and regulation of TAM. The Republic of Ghana is a low/middle-income country located on the West coast of Africa. It is a functioning democracy with a health care system that is governed by the Ministry of Health (MoH) in conjunction with other local and international governmental and non-governmental agencies. The TAM market and industry are regulated by the Public Health Act. Section 123 of the Public Health Act 2012 (Act 851) decrees about counterfeit drugs and herbal medicinal products: “*A person shall not manufacture, import, export, supply, possess or offer for sale a counterfeit drug, herbal medicinal product, cosmetic, medical device or household chemical substance.*” The regulation of the market of pharmaceuticals is exercised by the MoH and its agencies, including Food and Drugs Authority (FDA), Pharmacy Council and the Ghana Standards Authority (GSA). The FDA and GSA are responsible for certifying the quality and safety of all goods and products made in Ghana, including medicines. The TAM sector is regulated, promoted, and controlled by the Traditional Medicine Practice Council (TMPC) of the MoH. In addition, the government-founded Ghana Federation of Traditional Medicine Practitioners Associations is an umbrella organization that aims to ensure coherence and promote good practices among its members in Ghana. Currently, there are over 40,000 practitioners of traditional medicine in the organization. It is worth noting that Ghana's Centre for Scientific Research into Plant Medicine conducts research and develops traditional medicines. Since 1975, the outfit has been in collaboration with various traditional health practitioners [57]. While Ghana boasts of small and medium-sized pharmaceutical companies and despite WAM and TAM complementing each other, access to medicines is still problematic for emerging ailments caused by lifestyle and other environmental problems [7,9].

We targeted the 37 Military Hospital area (referred to as 37) and the Madina market, two busy suburbs of Accra as the empirical setting because of the convenience (easy to access) and the observed density of the population of buyers and sellers of both WAM and TAM. In the 37 suburb, we studied two herbal retail shops and four market vendors (who sell on tables). The area is a busy transit point for commuters to and from Accra central and its environs. One of the theoretically sampled herbal shops is situated in a filling station and the other is at a busy bus stop. The ‘bus stop shop’ has remained relatively the same in size and the amount of stock in the past decade but new varieties have been introduced over time. During repeated visits, it was noticed that customers come and go with great frequency, and they almost always engage in conversations with the seller about how helpful the medicines are and receive more advice on eating patterns and exercise, and prayer in some cases. The ‘filling station shop’ mainly sells imported and expensive herbal products. This shop is mostly patronized by the middle class who buy food supplements and other herb-based medicines imported mainly from the UK, China and elsewhere. The existing brand knowledge of such products concerns functional claims, process claims, and health symbols [58]. It must be emphasized that the place of origin is already an important attention grabber and convincing to customers. The shop owner had certificates of FDA (Ghana) approvals, municipal assembly, and internal revenue service certificates framed in glasses hanging on the wall. This reassures consumers of the shop's legitimacy and tax-paying status. This is the case for most herbal pharmacies that can easily be spotted in most city centres because they are in the public eye and not in obscure places or villages where the FDA is too stretched to extend pharmacovigilance.

On the other hand, Madina is a suburb of Accra known for its busy streets and open-space market. TAM vendors position their vans with large Public Address (PA) systems to appeal to potential buyers. The vans are permanently positioned in the area and are moved occasionally when there are construction works or a major clean up exercise by the municipal assembly. All four of the studied vans were claimed to be owned by doctors or businessmen with ‘self-proclaimed’ Dr. Titles. One company has two vans positioned in two different places (about 300 m apart) in the large market, selling mainly one or two products from the same entrepreneur. Their medicines cure similar diseases as observed from the long list of ailments printed on the adverting boards. They are in industry parlance ‘me-too’ herbal products (alternative products that solve the same problems as those on the market; cf. ‘me-too’ drugs that are broadly defined as “chemically related to the prototype, or other chemical compounds that have an identical mechanism of action” [59]. The two other street vendors in Madina on the other hand sell just about every type of TAM and WAM. We also interviewed the owners of two herbal shops in Madina's main shopping street. Our interviews centered on where they source their medicines, how much they know about the production process, what they think about the efficacy of the medicines, and what kind of complaints or feedback they have received from their customers. These questions were slightly modified for every shop owner/seller.

### 3.2 Study design and theoretical sampling

This study deploys a synthesis of longitudinal ethnographic fieldwork [60] that was carried out between 2011 and 2020, with multiple visits for observations. The fieldwork also included analysis of documents (brochures, official statements, and websites), semi-structures interviews and interviews using open and probing questions, and focus group discussions with vendors. We sought information about the local informal TAM market and WAM retailers with a vast array of shops and open market vendors (cases) in their real-life settings [61].

We first articulated the main objectives of the study and designed a fit-for-purpose informal approach to systematically collect data and undertake analysis that seeks to tease out what might be the optimal policies for fixing the longstanding problem of counterfeits. We examined the central issues, emergent themes, and recurring patterns that focus on the research question within the boundaries set in the research protocol. The objectives of the contribution were two-fold: first, we sought to determine the relationship between the problem of counterfeit medicines and the (health) institutional responses (policy, advocacy, education, and strengthening of the local pharmaceutical industry). Second, we underscored the specific co-factors (structural, market, and microlevel) which influence the consumption of counterfeit (or unregulated) WAM and TAM leading to market violence inflicted on communities.

The study is based on an interpretive (inductive) research approach. Noblit and Hare [62] define the mechanisms through which voluminous meta-ethnographic data can be aggregated and condensed to ensure that a research report offers cogent syntheses of the results (see Refs. [62,63]). Thus, the interpretive approach is not about hypothesis testing but a nuanced probing for insights from triangulated data in the marketplace and among policymakers and experts to inform policy. It is consistent with a post-positivist philosophy of science that relies on social constructionist ontology and epistemology. Here we seek contextually relevant meaning and truth from the voice of informants rather than generalizations. This study is inspired by the grounded theory approach to qualitative research [64] where theory is derived from data (subjective views of practitioners and observations).

### 3.3 Data collection

This section offers detailed information about how the different data sources were triangulated [60]. It also sheds light on how the institutions and participants were selected. The investigations conducted between 2011 and 2015 involved more formerly structured studies with agencies such as the FDA Ghana (previously Food and Drugs Board Ghana), various units at the MoH, the Pharmaceutical Society of Ghana, Customs Excise and Preventive Service, and GSA (previously Ghana Standards Board) as well as international agencies (see Supplementary Material for the complete list of the agencies and institutions interviewed for this study; part of the data have been published elsewhere, see Ref. [10]. Besides the interviews, these institutions provided brochures and other documents. Our interviews centered on explaining unclear issues in the documents and brochures as well as the current state of affairs of the counterfeit problem.

These institutions were not randomly selected, they were theoretically sampled. They were based on our initial interviews with two University Lecturers of Social Pharmacy who gave details of the appropriate institutions (with legislative mandates) involved in giving institutional responses to the phenomenon of counterfeit medicines. They also recommended experts to be contacted, so it was easy to know whom to approach for scheduling our interviews. The aim was to understand the institutional responses and the difficulties in fighting the counterfeit menace.

The insights drawn from the early phase of the study informed the later phase investigations in terms of the framing of the research question that specifically addresses the TAM sector. The approach used in the later phase of the study (2016–2020), however, is unlike the conventional approaches where letters are sent to interviewees prior to meetings. Informal markets require fitting informal investigative approaches given the risk of being perceived as an undercover police or FDA (Ghana) agent. Thus, we simply walk in as regular customers, purchase a cheap product, and engage vendors in friendly conversations (i.e., interviews). Equipped with a notebook and curious eyes we took the opportunity to carefully observe and listen to interactions between vendors and customers whenever a client came in and interrupted our interviews. The data for this study included 34 phenomenological interviews, carried out between 2011 and 2020 (see Supplementary Material, Table S1). The interviewees included ten customers, four owners of herbal/chemical retail shops, four open street market vendors with tables (in 37), and four with vans (in Madina). Additionally, we also interviewed two pharmacy owners and ten other industry experts of diverse backgrounds: a global health expert, a US Pharmacopeia expert, representatives of the Pharmaceutical Society of Ghana and Pharmacy Council Ghana, university researchers as well as officers of FDA Ghana, GSA, and MoH. Besides these interviews, we also witnessed and documented the sales pitch of WAM and TAM sellers in passenger/commuter vans (on five occasions in 2020).

### 3.4 Ethical considerations

Our study protocol ensured anonymity of respondents. All interviews with formal agencies were conducted after informed consent had been signed.

### 3.5 Data analysis

Our approach to data analyses follows but adapts the classic inductive content analysis [64,65]. We used this approach to decipher meaning from cues and clues in order to gain insights into the markets of counterfeits of TAM and WAM in the above research setting. The motivation for such an inductive approach includes flexibility (different from the rigidity in deductive approaches) and the epistemological advantage of the proximity to the context of human behaviour in a market setting. We make sense of the data by critically/analytically studying them in the quest to describe, name, and classify the data into discrete components. Such a process of data segmentation and meaningful descriptions is mostly known as open coding [66]. Here, we engage in textual analysis of triangulated data (observations, interviews, and documents).

The first step focused on the categorization and thematization of various elements of the interview data (the first-order categories). Here, no transcription was needed since the interview data consisted of field notes. Second, emerging themes from the above ‘sieving and separation’ of relevant data were aggregated and compared with the separate notes from the observations of various TAM/WAM product packages to produce second-order categories. Third, after studying the notes from observation and interviews, these were also compared with underlined issues from documents/brochures stating the mandates of the institutions and their responses to the current situation of the counterfeit issues on the market. Fourth, we identified similarities and emerging patterns across the triangulated data [60], after which we carefully evaluated and adumbrated emerging issues based on a hierarchy of relevance. In this phase, we assembled a constellation of meanings from the various sets of data. This allowed for easy interpretation.

Fifth, there was then an iteration between (back and forth visiting and revisiting) all the above phases and writing the first draft of a narrative. This approach is also known as an abductive approach where the researcher goes back and forth between data sets and extant literature. This process subsequently entails the iteration of the triangulated data with extant literature to ensure consistency, rigour, and robustness of synthesis of our interpretation of the data vis-à-vis state-of-the-art institutional and market dynamics of the phenomenon under investigation. Sixth, in so doing, we return to the central objective of the study by searching for depth and underlying co-factors that determine the demand and supply of counterfeits as stated in the objectives as well as how institutional responses as stated in the documents help answer the questions.

In the next section, we deploy a descriptive synthesis (a condensed version) of this ethnographic fieldwork marked with a few quotations by respondents to make a point when necessary. Through a narrative that articulates the nature and structure of the informal market (mainly TAM, but also WAM), we explain why there are no rigid differences between WAM and TAM in terms of the underlying reasons why they both require explicit political strategies and policies in the quest to protect consumers. Our discussions place major emphasis on the depth of multiple voices and insights into the locals’ construction of the institution of the informal market rather than large-scale statistical averages and inferences for the sake of percentages.

## 4 Findings

The section offers a thick description of the following: the nature and structure of the TAM market, the demand and supply side of counterfeit WAM and TAM, destructive and unproductive enterprises in the WAM and TAM markets, forms and nature of counterfeits, and institutional responses to the counterfeit problem. The study identifies three major forms of counterfeits: (i) over-the-counter (OTC) drugs – i.e., non-prescription medication from legitimate pharmacies and dispensaries that have been infiltrated, (ii) over-the-net (OTN) drugs – e.g., antibiotics, obesity drugs, or Viagra (male erectile dysfunction drug) – bought on the internet stores, and (iii) open-street-market (OSM) drugs and traditional (mostly herbal) alternatives. Further, the study found that TAM on the market may not be adulterated per se. However, five fundamental characteristics of the sector announce a threat to consumer safety. First, there is no scientific basis for many of the claims of efficacy made by vendors who are not registered with the FDA (Ghana). Second, the manufacturing process for a good number of products takes place under conditions where safety and quality cannot always be guaranteed since the operating standards are without regard for Good Manufacturing Practice (GMP). Third, the side effects of the medicines are mostly unknown due to their (officially) untested nature. Fourth, the sheer number of producers and sellers complicates any efforts to control the sector—and that is a disconcerting situation. Fifth, the numerous versions of TAM (tablets, syrups, and bitters, a combination of tree barks, etc.) and the informal markets (within which the demand and supply of counterfeits or unregistered TAM occur) make it difficult to mitigate any value destruction activities that constitute market violence.

### 4.1 The nature and structure of traditional and alternative medicines market

In what follows, we synthesize the current nature of the demand and supply of TAM in Ghana. The most significant sources of danger are divided into two domains, namely the demand side and the supply side. Among the most prominent features of demand-side issues are the perennial lack of access to quality drugs from secure chains on the market at affordable prices. Low-income status plays into this analysis. However, there is also a problem with the market which consumers struggle with: the ignorance of the difference between evidence-based drugs and toxic substances in beautiful packages (not even experts can always figure out the differences). Additionally, the lower prices of such products as well as the perceived quality given the shiny packaging deceives many to patronize such goods. The above leads us to certain social questions, such as unethical consumption, peer pressure, and self-medication. These can be seen as socially accepted ways of seeking an alternative cure in the absence of viable health infrastructure and well-controlled pharmaceutical markets. Thus, the underprivileged are forced to accept alternatives that are offered on the market. Finally, deceptive advertising by sellers and street vendors, and even some clinicians contribute to the demand for counterfeits.

On the supply side, various market and technological factors explain the reasons for the proliferation of counterfeit WAM and TAM. These include (i) easy access to manufacturing/sales/packaging technologies; (ii) lapse regulatory systems that allow the direct-to-consumer marketing of such products; (iii) and underserved market where destructive entrepreneurs find a way to fill the gap with unapproved alternatives. Thus, unlimited supply meets great demand. Suppliers of counterfeits mainly consist of illegal/unregistered organisations of WAM/TAM entrepreneurs and herbal medicine preparation ventures (HMPV). By HMPVs, we are referring to very small scale or sole proprietary manufacturers and distributors of herbal preparations, bitters, and mixtures that are sold e.g., in lorry stations, streets, and open markets. The open market vendors do direct-to-consumer marketing where the immense threat comes from. This is due to the weak formal control of pharmaceutical value chains [67,68]. The sophistication in the supply of counterfeits is made possible due to the availability of technologies to criminals. This is a global issue. In the USA for example, 97% of all online drugs are counterfeits or at least without the US Food and Drug Administration's approval [18].

Anker et al. [58] provide a framework for health branding that is useful for our analysis: it includes functional claims, process claims, and health symbols and obscuring of side effects. The powerful subliminal approach works even if key abiding questions remain about manufacturing and processes and active ingredients of street vendors' offers. As symbolic gestures, prayers are often offered, and this seems to have the psychological effect of removing doubts. Pricing and convenience are the main reasons why consumers patronize herbal mixtures of all sorts. Religion and faith are mixed with the science of curing. The cure is regarded as a question of a need for appealing to the metaphysical that will induce supernatural powers for medicines to work, including holy water from pastors. These ‘holy healers’ are among the main competitors of the pharmaceutical industry even during the COVID-19 era. The government and FDA Ghana have gone to great lengths to debunk false claims (scientifically unproven) by discouraging consumers from purchasing such goods. Self-medication and the belief that WAM contains too many chemicals or that they are stored improperly are among the reasons why people buy TAM versions.

A typical scene from a street salesperson (mostly men) can be articulated as follows: He courteously greets the passengers; he requests to say a word of prayer and proceeds with well-rehearsed, polished rhetoric about the importance of your health, why his medicine is the best and why you need it now and not later. Hardly are any questions asked. He tells them the price and people stretch their hands with cash in exchange for the medicine. It works for some (placebo effect?), but it does not work for everyone. The observations now demonstrate that we understand too little about the demand and supply of counterfeits worldwide although the menace is on the rise. More importantly, within the demographic composition of Ghana, people of all demographic classes are equally affected, regardless of age, gender, and income level according to experts. The reason for this is that the medicines have infiltrated the supply chains given the overstretched resources of institutions to control all the entrepreneurs who engage in the sales of TAM as a form of necessity entrepreneurship.

**Fig. 1:**
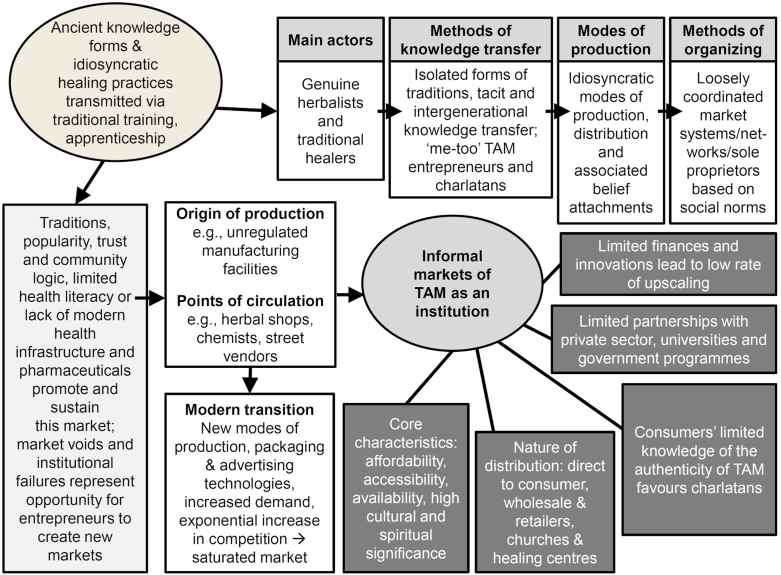
The origins, nature, and structure of the informal market of traditional and alternative medicines (TAM).

The formal institutional structures however make the greatest difference in protecting supply chains. Many street vendors and chemists (shop owners) echo the fact that the common challenges faced by legitimate entrepreneurs are production technologies, finance, and know-how. This means that structurally they have problems scaling up as registered microenterprises. They compete based on direct-to-consumer marketing, availability, proximity to consumers, and predatory pricing compared to conventional medicines. See Fig. 1 for a condensed representation of the origins, transitions, metamorphosis, and current modes of circulation of TAM.

### 4.2 Destructive and unproductive enterprises in the markets for western allopathic medicines and traditional and alternative medicines

Increasing affluence alongside the persistence of low-income households [69] has meant that emerging and existential healthcare needs are hard to meet. Consumers demand remedies for new pathologies that are causing high levels of morbidities and even mortalities across Africa [11,70]. Formal or informal control of this market is either limited or exists only in the books. Generally, such market actors are unregistered with public authorities. Moreover, they do not pay taxes and are therefore able to evade any form of regulatory control. Their substantial number and diverse modus operandi make it more difficult to control them. This also means that they compete away customers from authentic pharmacies or genuine herbalists.

Further, in the street markets for medicines, apart from the tangible products and real services (such as prayer), there is also a market for the appeal to the metaphysical. This brings in innumerable types of players and explains the complexity of the sector. ‘Holy healing’ is real even when it mostly ends up tragically for those who follow such without seeking proper medical attention. Healing water and herbal potions without any guarantees or background checks are flourishing on the market even during the challenging times of the COVID-19 pandemic [[71], [72], [73]]. The activities of such so-called holy men include the spiritualization of cure in response to the institutional logics of the market environment. The phenomenon is old. Etymologically, ‘sorcery’, translates as *pharmakeia* in Greek. This is how the term *pharmacy* was derived. “*Diviners, enchanters, witches, and sorcerers employed drugs and other potions to put them or their clients ‘in the spirit’ so their ‘magic’ would work. The drugs, then, came to stand for sorcery of all kinds*” [74]. This ancient practice has only morphed into two distinct practices in the same industry: (i) tried-and-tested TAM or formalized, rational drug production and (ii) spiritual healers acting as doctors using herbs. Hence, the value destruction activities come in the form of deceptive marketing, righteous devils or charlatans professing ‘pious lies’ as they pose as doctors to deceive consumers. These transactions are based on a major factor: cautious hope—even in the unseen.

Unregistered pharmacies, TAM producers, and street vendors have closer proximity to consumers. This is an important source of appeal. In marketing theories, relationship marketing is an important source of consumer value co-creation. This happens especially because of the experience consumers derive from being heard and being able to receive frequent attention from sellers [10,75]. Consumer interaction with the TAM manufacturers and distributors, therefore, makes them both co-producers and even co-creators since it is their timely and consistent feedback that helps producers to innovatively modify their offerings to attract more customers [76,77].

Additionally, many consumers do not perceive WAM as conventional or orthodox. If anything, TAM is orthodox and WAM are the new medicine, whether produced by the local pharmaceutical industry or imported. For the past half-century, both types of medicines have been consumed side-by-side with each other; (see for example [12]). There are also licensed chemists who sell TAM alongside WAM. We are not suggesting that TAM pose any significant threat any more than WAM. On the contrary, traditional medicines are even highly trusted as the only cure for certain tropical pathologies that lead to high mortalities and morbidities. To persuade the West African consumer about this much requires a lot of convincing.

### 4.3 Main distinguishing features of observed medicines

A different approach would be to take samples of TAM and WAM to analyse their content and/or test their efficacy in a laboratory (see Ref. [5]). Ours however is a socioeconomic approach where the study is not directly about the active pharmaceutical ingredient (API) content of medicines but the market context within which exchanges unfold. In the list below, we account for the audit of the common features of OTC and OSM medicines after observing different popular herbal syrups, and tablets meant for various ailments. We had documented clues that led us to suspect that such medicines would not have possibly been approved by the FDA for sales because certain external characteristics of their packaging make their integrity questionable.

Red flags indicating the need for caution:

• Most of the medicines had no indication of contents.
• Some etiquettes were fading off after having been kept for so long in the sun.
• No indication of possible adverse effects.
• An absurdly lengthy list of different diseases that are cured by one medicine.
• Expiry dates are either inexistent or not always visible.
• Spelling mistakes on instructions as indicators of carelessness and questionable quality and professionalism.
• Superstition and subliminal appeals are integral parts of the marketing strategy. When asked where the shop owners had their training, at least three of them stated that they have learned their trade through visions and dreams. Others also said they have learned the craft from their grandparents and parents. The latter is plausible, but the former is hard to verify empirically. Five of the open street vendors argue that they are just resellers who purchase from wholesalers ‒ just business.

Consumers are either oblivious to the dangers or they intentionally ignore the red flags that may be warning them against the potential dangers. We inquired politely about how well the consumers were patronizing their goods, how sellers can differentiate good medicines from bad ones, and whether the shops are registered. We received intriguing answers. The sellers also receive feedback from customers. A street vendor (female) argued angrily when we enquired how she differentiates between good and bad herbs and bitters from her wholesale. “*They [wholesalers and customers] tell me what the medicine does, and I learn it, I just buy more, and resell. There is no fake in herbal medicine. If it does not work, it means it is not good for you.*” Three friends standing by with their goods nodded their heads in agreement. Another vendor added, “*one only needs to change it or get rid of it and buy from somewhere else. Hardly do we report this to the police. It is how the informal market works because going to the police is a waste of time”*. We coded this as how vendors personalize medicines based on consumers’ feedback. Vendors also depend on word of mouth and testimonies of satisfied customers to advertise their products. In our presence, a police officer in a uniform came to purchase a product and profusely praised it. This shows that some claims are true, but charlatans are manifold, too.

Some sellers of TAM for penile dysfunction add Viagra to their herbal concoctions, making patients believe that such medicines are efficacious. What this does is that it affects the trust in TAM (*Pharmacist 1, Accra mall*). Although some extent of damage is done through the sales of certain TAM products, it is difficult to pinpoint the quantitative extent of damage due to Africa's statistical tragedy [78].

This is not to suggest that all producers do act with criminal intent. “*Some of these sellers just do not know any better and in some cases, they have no alternative economic route of escape from economic distress*”, says an expert from the US Pharmacopeia in an interview in Washington DC (2013). “*Some of the medicines are also knock-offs. They are not original, but they are not necessarily dangerous*”, says another Pharmacy expert from Pharmaceutical Society of Ghana. “*Unlike those peddling traditional medicines in the streets of commercial centres, in the villages, they do have genuine intentions to heal the patients. They mostly have no profit-making intents, and they live among the people with whom they have a long history of family ties*” (University researcher 1). “*Their medicines seem to work but we still need to scientifically verify them; but resource constraints remain our major problem*” (University researcher 2). The university researchers further describe how necessity entrepreneurship makes it difficult to enforce control mechanisms to curb counterfeits and spurious drugs made by quack scientists.

A mammoth amount of both WAM and TAM for common ailments are easily accessible across Ghana and more specifically in the two observed suburbs. Interviews with ten frequent users of TAM demonstrate that consumer confidence is high and hardly do they question the source, the potential danger, or the integrity of actors along the supply chain. For example, eight of these consumers have bought TAM or WAM on the open street market that did not work. They got adverse reactions, but they did not report those to the authorities or the doctor. Thus, consumers’ romanticism with a blind trust is high and the questions about the values of sellers take a back seat.

### 4.4 Institutional responses to the counterfeit problem

A key finding here is that in the course of this longitudinal fieldwork, there have been major encouraging milestones with respect to structural changes to the regulation of the informal and formal markets of TAM and counterfeit WAM. More precisely, during this study, both the Ghana Food and Drugs Board (FDB) and the Ghana Standards Board were renamed as Authorities (FDA and GSA, respectively): giving them more power to regulate the WAM and TAM market (demand and supply). Despite these major improvements, they have limited capacity in terms of human resources, finances, and infrastructure to tackle the overall scope of the problem of fakes and unregistered products in the system. As the head of FDA put it, “*the political will exists, but we are very limited in what we can do, even here in Accra alone. It is not easy to cover everywhere. With the backing of the police, we have been embarking on raids in various quarters of Accra when we get tip-offs*”. The head of Standards Authority continues: “*Our main challenge now is formalization of the informal market (to get entrepreneurs to register their products and get approval before they start selling)*”. Additionally, we also witnessed how TAM gained prominence as the MoH approved certain TAM for use in Ghanaian hospitals. For example, the procurement division of the MoH in Ghana has started allowing approved TAM in hospital dispensaries (one of the authors was present in the office of the Director of Procurement when the list of approved TAM for use in hospitals was submitted to her in 2012. Someone knocked on the door, came in, and handed a file with the list of approved TAM. “*You are lucky today*”*,* she said, “*exactly what we are talking about. The government is doing its best to mainstream our traditional medicines because they are very efficacious in solving some of our common health problems*.”

Moreover, over the years, there have been systematic and sustained efforts by Ghanaian universities to introduce courses in Pharmacognosy in the quest to formalize the informal sector of traditional medicine practice. The word ‘Pharmacognosy’ has a Greek origin; ‘pharmakon’ means ‘drug or ‘poison’ and ‘gnosis’ means knowledge. The American Society of Pharmacognosy defines Pharmacognosy as “the study of natural product molecules (typically secondary metabolites) that are useful for their medicinal, ecological, gustatory, or other functional properties”.

The above indicates that there are institutional responses to mitigate the proliferation of counterfeit drugs and aid the TAM industry. Those responses, notwithstanding, fall appallingly short to counter a problem of this magnitude. Ideally, these responses must include robust public health funding, efficient administrative systems at cross-sector levels for reducing waste, and an increase in human resource training in the sector. On the industrial front, more subsidies for the development of SMEs will be constantly required to upgrade the sector to ensure scaling possibilities. Part of the problem is that the mainstream responses to WAM counterfeits are official, documented, professionalized, and formalized. These are either by organisations or private enterprises such as mPedigree [79]. By this, we mean that such interventions target only industrially made drugs that are labelled, coded, serialized, and are thus traceable. A prime example is the recent European Union (EU)-wide directive to falsified medicines regulation that requires that “*… market authorization holders...will have to ensure that every prescription pack of medicine that is circulated in the EU market has a Unique Identifier, a two-dimensional matrix bar code, and a human-readable version* …” [80]. This is not the case with the markets for TAM. Unfortunately, there are as many consumers of TAM as there are of WAM.

The FDA and other agencies in Ghana are making good on their promise of cleaning the market of fakes but their stretched resources make it a struggle to contain the menace. There are additional major efforts in place to mitigate counterfeits in Ghana. There is for example a sophisticated centre established by the US Pharmacopeia that seeks to ensure drug quality in Ghana and the larger West African region. In addition, the WHO IMPACT (The International Medical Products Anti-Counterfeiting Taskforce) [81] is intervening, but they hardly reach the informal supplies and demands. However, our observation demonstrates that while formal interventions are useful, they are less effective when they lack the required financial resources.

“Yes, their office (TMPC of MoH) is there, but they can’t reach all of us because they are not functioning well, and we are also very many. They need a committee that will organise itself and reaches all of us. In that way they will know how to help us because our medicines are powerful. This tree right here can take care of the whole of Ghana if we invest in it because it can cure many diseases”, claims a TAM seller (37 area, Accra).

There is an observed disconnect between the market and formalized control systems. The sector is believed to be thriving, but producers lack technologies and finance. “*My license has expired but this FDA (Ghana) people take a long time to help us. But if someone brings any product from China, India, or somewhere they immediately get their licenses and approvals...And we who are here, we are suffering*” (a TAM dealer). Our study finds no objective data (from the Ghana Statistical Service) on the quantity of drugs that are seized per year by the FDA or Customs Excise and Preventive Service (CEPS), neither are there any data on any dimension of the phenomenon of counterfeits. There is neither information platform nor a systematic study that offers a strong basis for serious policies. This means that there is still so much to be discovered about the complex, multifaceted, and countless dimensions of the informal medicines’ market. For example, knowledge exchange occurs mostly at the market level where sellers and producers coy the latest trend in packaging and advertising techniques even using television and radio. After inspecting over 50 different offerings on the market, a worrying trend was discovered; many included Chinese packaging and carefree English translation but no noticeable approval number from the FDA Ghana.

## 5 Discussions

### 5.1 The co-factors that explain the proliferation of counterfeits

There is emerging evidence that explains the rise of fake WAM and unregistered TAM. All these co-factors are further elaborated below. These five major interrelated issues point to the need for urgent institutional responses: The first explanation is institutional in nature. There is a persistent institutional weakness to control the menace, hence, its exponential increase. The institutional weakness also refers to three interrelated issues: poor public health services, lack of access to medicines and the high cost of WAM. Patients in low-income nations pay many times more for their generic medicines than those in the West. In response, a need is created, and destructive entrepreneurs see an opportunity to profit from this market vacuum. This intersecting role of consumer and producer leads to what Humphreys and Grayson [77] refer to as co-production and co-creation. However, this enlists consumers in market violence [48] against themselves; leading to the loss of money, purchase of toxic medicines, or those that lack active ingredients. Part of the reason consumers buy suspicious medicines lies in a little of all the above discoveries.

Second, apart from the informal and unregulated nature of the market, the rise in necessity entrepreneurship (a product of the lack of economic opportunities in emerging economies) along with the availability of easy-to-use packaging and advertising technologies have made the TAM sector a major competitor of WAM that can hardly be ignored. These technologies are cheap, easily accessible, and do not require complex technical training to use them. Necessity entrepreneurship is pushing unscrupulous individuals and groups to offer dangerous versions of good medicines. This is generalizable to other low-income countries. Nevertheless, necessity entrepreneurship must also be broadened. It goes beyond the socio-economic sphere. It is also found in the political and religious fields and in pure crime syndicates – wherein lie the main competitors of the pharmaceutical industry.

Third, the informal markets of TAM are structured in ways that allow them to evade formalized interventions and regulations. Such interventions hardly respond to the institutional logics in which these informal markets operate. For example, at the same time as the situation worsens, more is being done by retailers to subliminally dampen the fears and outrage of informed consumers through the creation of unnecessary medical needs. This is done through new direct-to-consumer advertising technologies and perversely convincing word-of-mouth. Examples of these include weight loss and sexual dysfunction remedies and food supplements. It is argued, therefore, that a strong focus on curtailing WAM counterfeits is a partial diagnosis of the problem: lack of access and affordability of medicines stemming from a weak pharmaceutical R&D and manufacturing base and under-resourced healthcare infrastructure remains a challenge. The general problem of unemployment must be addressed with urgency since the unemployed seek asylum in the informal pharmaceutical markets.

Fourth, standardization allows entrepreneurs to derive advantages from economies of scale and reduce production costs allowing the sector to flourish with little economic risk while inflicting violence on consumers. One can claim that the volume and profitability of the counterfeit business is greater than non-counterfeit and branded ones. Where is the evidence? It is seen in the number of buyers and sellers that keep increasing over time as we observed in the street shopping centres across the cities in Ghana. Where then is the counterevidence against that? No one has it either. Fake and spurious medicines straddle two worlds: formal and informal markets. They are inextricably intertwined, yet worlds apart in their effects and efficacy and the conditions under which they are prepared. The imitation of orthodox drugs naturally leads to limitations in their efficacy, but the public patronizes them anyhow. So, why then do counterfeits thrive? Most importantly, there is a high demand due to the nature of the climatic and environmental conditions in tropical regions, irresponsible lifestyles, and unhealthy consumerism leading to high disease burden—worsening the social determinants of health [82]. Here, the lack of access to quality medicines, the high cost of branded medicines, and even the availability of counterfeit medicines make the situation a bit more complicated. Fifth, personalization and co-creation of medicine with patients have the psychological effect of increasing consumer confidence. This makes consumers complicit in the market violence against themselves.

If the demand for potentially unsafe medicines is exponentially increasing, then it can be explained by ignorance, poverty (the prices offered by these sellers for antimalarials are generally good), and lack of awareness about the potential danger of these drugs. Certain internalized cultural practices and hedonistic and unethical consumer behaviour also contribute to the growth of this market. Technological change and increasing affluence on one hand and increased disease burden alongside persistent poverty on the other have led to high consumer demand for medicines that are deemed ‘chemical free’; Table 2 offers a global overview of the reasons for the demand for fake medicines.

**Table 2:** Reasons for the increase in the global demand of counterfeit medicines.

Characteristic of emerging economies	Characteristic of advanced economies
Lack of better choice/access to medicine	Easy access to dark internet; misinformation about the efficacy of traditional and alternative medicines (TAM)
Lack of access to modern health care infrastructure	Power of advertisement and blind trust in the internet vendors
Slow process in integrating traditional and conventional medicine into hospital dispensary systems and supply chains	Addiction to opiate pain killers and a high demand for drugs for the treatment of erectile dysfunction
Convenience: Sellers come to you with the promise. Their marketing techniques and proximity, follow-up, and encouragement work as a strong marketing strategy (emotional appeal). Prices are mostly lower and negotiable.	Emerging necessity entrepreneurships in advanced countries since the economic downturn: a shift from traditional underground drug business to pharmaceuticals
Difficulty in identifying the authenticity of packages and bottles	The flood of health-related information on the internet; trend towards self-medication
**Common to both developed and advanced countries**	
• High cost of medicines • Consumer ignorance/unethical purchasing • Increasing disease burden on households • Emerging lifestyle change, eating patterns, and quest for weight loss and erectile dysfunction medicines • Urbanisation, congestion, sanitation, and over-pollution related diseases	

### 5.2 Towards feasible technology and innovation agenda for consumer co-protection

“For the man with a hammer, every problem looks like a nail”.

**Fig. 2:**
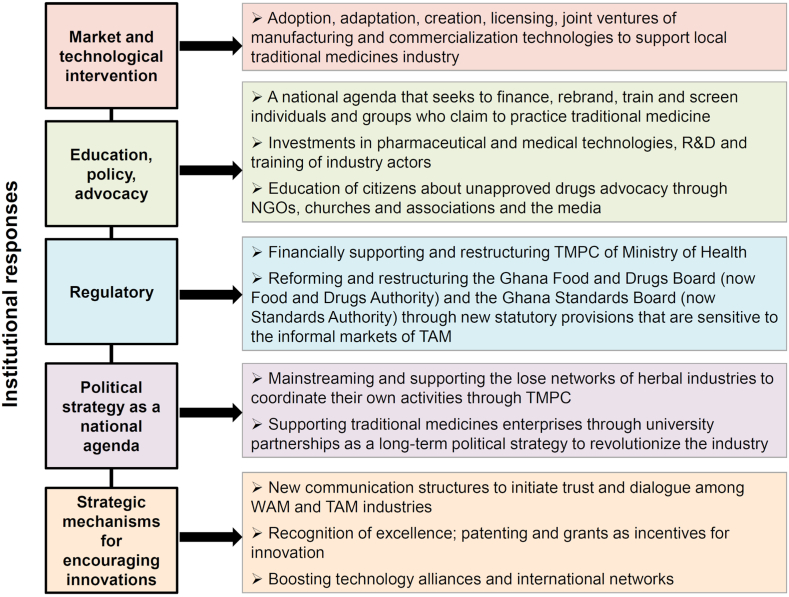
Coordinating mechanism as institutional responses. R&D – research and development; NGO – non-governmental organization; TMPC – Traditional Medicine Practice Council; TAM – traditional and alternative medicines; WAM – Western allopathic medicines.

Policies by government institutions, policies influenced by private and hybrid organisations, and even think tanks within the healthcare sector [83] must always be about WAM. That is not incorrect, but it may be incomplete and less innovative response to the central problem of patient safety. This is because policies are needed but they are not self-reinforcing. Second, some policies are simply not feasible in reality. Third, policies that ignore the informal market only answer a part of the big question of how to guarantee patient safety. Pharmaceutical counterfeiting is a burning issue that transcends national borders and is a hot topic that warrants attention from scholars and policymakers. It is an extraordinarily complex technical problem that requires a better understanding of the informal production and circulation of medicines on one hand and stringent pharmacovigilance, post-market surveillance, and education on the rational use and purchase of drugs on the other. Hence, this contribution matters in finding a contextually relevant market and institutional solutions on one hand and developing novel innovative strategies that affect consumer purchasing behaviour around counterfeit medicines on the other. This contribution sought to direct attention to the priority areas for immediate action: (i) to induce policy change on the local-global linkages for consumer co-protection and (ii) to offer policy guidelines as to the process of adaptation to the emergent questions on consumer safety in global health and how to be strategically well-positioned to answer these questions through scientifically derived information, novel technologies, and decision making. More specifically, the added value of this contribution is that it directs attention from the formalized forms of consumer protection that concentrate on the WAM. The proposed focus is on a more comprehensive and ambidextrous approach that seeks patient protection from all questionable medicines. However, the mechanisms for such controls may clearly differ from one context to another. Since resources for waging a war on both fronts simultaneously are scarce, a soft approach that seeks to educate both consumers and producers, and a hard approach where formal, graduated sanctions are applied, are needed. TAM producers must by law register in the traditional medicine producers' associations where the government's financial and technical support can be obtained. Consumers must be encouraged to question what they buy and buy only from licensed stores. Here in Fig. 2, we supply cross-sector coordinating mechanisms as institutional responses for co-protecting consumers.

The importance of TAM industry in saving lives cannot be underestimated [1,11,57,84]. Although TAM are not a panacea for every pathology, they probably hold the best promise for economies with high disease burden. This will be possible only if more resources were earmarked for the sector to create a more coordinated and properly regulated manufacturing and sales base. This means that global health actors (managers, policymakers, and clinicians) create the maximum social value with and for consumers/patients through strategy innovations for patient co-protection against counterfeits by gradually changing the institutional logic that increases the supply and demand of counterfeits. What needs to be studied further is the avoidable common ailments. It is conjectured that the public understanding of public health is extremely limited. There are still substantial portions of the population that are semi-literate or ‘miseducated’ about the social determinants of health, which leads them to concentrate only on inappropriate cures rather than prevention. The counterfeit phenomenon is also very much the phenomenon of TAM. They are both as old and complex. Yet, whether there will be shifts from the use of approved medicines to consensual acceptance of holy fakes and fancy poisons remains to be seen. Further, the magnitude of the problem requires robust interventions, which to a considerable extent are lacking on the part of African governments [85]. This means technological mechanisms are required to scale, boost, and accelerate the industrialization of local TAM entrepreneurial firms into meticulously organized structures. As a growing number of works suggest, the creation, adaptation, or adoption of technologies [86] can potentially activate Africa's industrial potential while strengthening entrepreneurial development [87]. It is no news that the lack of technological advancement is what distinctively separates Africa from the other continents. It is therefore argued that any policy or effort aimed at mitigating the problem of counterfeits must be pragmatic, innovation-driven, and ambidextrous to be successful. Most importantly, we emphasize that the whole discussion surrounds consumer or patient protection even in these challenging times of COVID-19.

### 5.3 Limitations of the study

Despite the depth of information gathered, we did not seek to make any sweeping generalizations. Rather, we sought to gain profound insights into the sector to offer evidence-based policy innovations and to suggest new frontiers for further investigation. The phenomenon under investigation is highly complex and this study only offers a preliminary understanding of the issues. The study does not even cover major cities in Ghana, let alone countries across Africa. Additionally, it does not specifically study specific medicaments or TAM but random observations of both and how they circulate on the market. We reckon that our conclusions give a wider view of the market although a lot still needs to be studied about specific institutional changes that are emerging within the sector especially as the pandemic exacerbates various health conditions.

## 6 Conclusions and implications

This study is based on ethnographic fieldwork and answers the question: *How can global health actors (managers and policymakers and clinicians) create the maximum social value with and for consumers/patients through policy innovations for patient co-protection against counterfeits/unsafe drugs?* The overriding factor in this study is that the manufacturing and sale of counterfeit medicines (TAM or WAM) have enormous implications for public health outcomes. An investigation into the role of destructive entrepreneurship and market violence through the sales of these products is not to underestimate the importance of the sector. However, the study sought to highlight the abuse in the sector through evasive or criminal forms of entrepreneurship vis-à-vis the possible institutional responses to sanitize the sector. We conclude that overall, approaches to global/public health governance pay little attention (limited investments) to the complex economic gamut of TAM. This, in whole or in part obscures the sectors’ special significance and undermines overall public health.

Market violence is made possible through the weaponization of consumer ignorance by destructive entrepreneurs and through outright deceit (functional claims, process claims, health symbols, and obscuring of side effects), weak regulatory control, or too-light punishments for offenders. Moreover, after identifying the nature and structure of the informal market for WAM and TAM, it has become clear that the root of the problem within this sector lies in economic development, health systems, and the general inequalities, namely: lack of access to quality and controlled medicines and lack of opportunities for entrepreneurs.

In three ways this study makes contributions to the theory of destructive entrepreneurship and market violence within the context of the pharmaceutical industry in emerging economies. First, entrepreneurship can be both destructive and produce violent outcomes. However, in many cases, the entrepreneurial intention is not to cause harm but to make a living. First, this is because most street vendors have little knowledge about the production process of their merchandise. They are mainly interested in retailing to make some income. Second, weak socio-economic and institutional structures and health systems and a struggling pharmaceutical industry open the way for alternatives that may not always produce the desired health outcomes. Third, over time, the growth of such destructive entrepreneurship seriously undermines trust in legally operating businesses and public health institutions. There are structural/institutional issues that set limits on the extent to which the TAM and WAM pharmaceutical SMEs can realistically develop the industry to contain the phenomenon of counterfeits. The fundamental problem can be ascribed to something much deeper than the mere lack of resources or absence of finance for the burgeoning industry; the lack of political will to secure total independence for public health through the harnessing of national medico-technoscientific, industrial and market resources, and the provision and accessibility of primary health care that is properly regulated to ensure that the supply of both TAM and WAM are of the highest quality.

A final nuanced explanation is that serious policy, market, and public health interventions are needed because consumer awareness about the dangers is also low. Affordable value propositions by such enterprises keep growing in sophistication to meet the needs of consumers. In the long run, such problems become insurmountable. The study problematizes the structure and nature of the TAM sector and determines the various co-factors that affect the way the market operates and what hampers change. In the quest to ensure patient safety from counterfeiters, we explain how to deploy an ambidextrous approach of both hard (technical and formal) and soft (social and educational) proactive interventions for WAM and TAM.

### 6.1 Policy recommendations — caveat emptor

The findings suggest that the use of TAM is as widespread as the use of WAM among all income levels and not only among low-income households as previously thought. That shows that an increase in demand has led to an increase in unscrupulous suppliers who compromise drug quality on the market. The effects are massive, and their violent proportions are still understudied. A new architecture of both industrial investments in local pharmaceutical and TAM industries is needed along with policy solutions for consumer/patient protection. Shivarajan and Srinivasan [88] refer to this as ‘global knowledge networks through trust-based partnerships’ among neglected consumers and businesses and non-business actors for creating the maximum social value for consumers. Above all, intensifying campaigns at all levels about the dangers of unsupervised self-medication and blind trust in retailers whether online or on the street is highly recommended as summed up above in the Latin phrase ‘caveat emptor’— buyer beware.

## Author contribution statement

Frederick Ahen: Conceived and designed the experiments; Performed the experiments; Analyzed and interpreted the data; Wrote the paper.

Kwame Ohene Buabeng: Conceived and designed experiments; Contributed reagents, materials, analysis tools or data.

Outi M. H. Salo-Ahen: Analyzed and interpreted the data; Wrote the paper.

## Funding statement

F.A. received financial support from the following foundations: The Wallenberg Foundation, the University of Turku Foundation, Stiftelsen för Handelsutbildning i Åbo and the Foundation for Economic Education. The Article Processing Charge (Open Access fee) was catered by Gösta Branders research fund, Åbo Akademi Research Foundation. None of the funding bodies had any role in the design of the study, collection, analysis, or interpretation of data, or in writing the manuscript.

## Data availability statement

Data included in article/supplementary material/referenced in article.

## Declaration of interest's statement

The authors declare that they have no known competing financial interests or personal relationships that could have appeared to influence the work reported in this paper.
