# Supplementary material for: Market violence through destructive entrepreneurship: Assessing institutional responses to the proliferation of counterfeit traditional and alternative medicines in Ghana
Source: Heliyon. 2023 Feb 21;9(3):e13881. doi: 10.1016/j.heliyon.2023.e13881 (PMC9988503; doi:10.1016/j.heliyon.2023.e13881)
Supplement: Multimedia component 1 [file mmc1.docx]

**Supplementary Material**

#### Market violence through destructive entrepreneurship: assessing institutional responses to the proliferation of counterfeit traditional and alternative medicines in Ghana

***Frederick Ahen^a,#^, Kwame O. Buabeng^b^, Outi M. H. Salo-Ahen^c,^****

^a^Turku School of Economics, University of Turku, Turku, Finland

^b^Kwame Nkrumah University of Science and Technology, Faculty of Pharmacy and Pharmaceutical Sciences, Department of Pharmacy Practice, College of Health Sciences, Kumasi, Ghana; E-mail: [kobuabeng.pharm@knust.edu.gh](mailto:kobuabeng.pharm@knust.edu.gh)

^c^Åbo Akademi University, Faculty of Science and Engineering, Pharmaceutical Sciences Laboratory (Pharmacy) and Structural Bioinformatics Laboratory (Biochemistry), Turku, Finland; E-mail: [outi.salo-ahen@abo.fi](mailto:outi.salo-ahen@abo.fi)

^#^ Present affiliation: Åbo Akademi University, Faculty of Science and Engineering, Pharmacy, Turku, Finland; E-mail: [frederick.ahen@abo.fi](mailto:frederick.ahen@abo.fi)

* Corresponding author.

E-mail address: [outi.salo-ahen@abo.fi](mailto:outi.salo-ahen@abo.fi) (O.M.H. Salo-Ahen)

**Full list of agencies studied for this research 2011-2020**

**Ghanaian institutions**

- Customs Excise and Preventive Service (CEPS; <https://gra.gov.gh/customs/preventive/>)
- Ghana Food and Drugs Authority (FDA Ghana; <http://www.fdaghana.gov.gh>)
- Ghana Standards Authority (GSA; <https://www.gsa.gov.gh>)
- Ministry of Health (Procurement; <https://www.moh.gov.gh/procurement-supply/>)
- Ministry of Health (Traditional Medicine Practice Council, TMPC; <https://www.moh.gov.gh/traditional-medicine-practice-council/>)
- Ministry of Health (Pharmaceutical Services; annual report from 2007: <https://www.pharmacy.gov.my/v2/sites/default/files/document-upload/annual-report-2007.pdf>; current description: <https://www.moh.gov.gh/technical-coordination/>)
- Pharmacy Council Ghana (<https://pcghana.org>)
- Pharmaceutical Society of Ghana (PSGH; <https://psgh.societymanager.org/home>)
- Kwame Nkrumah University of Science and Technology Faculty of Pharmacy & Pharmaceutical Sciences (<https://pharmacy.knust.edu.gh>)
- Ghana Statistical Service (GSS, <https://www.statsghana.gov.gh>)
- Ghana National Malaria Control Programme (NMCP, <https://ghs.gov.gh/national-malaria-control-programme-nmcp/>)

**Multilateral institutions**

- US Pharmacopeial Convention (<https://www.usp.org>)
- INTERPOL (<https://www.interpol.int>)
- WHO (<https://www.who.int>)
- The Global Fund (<https://www.theglobalfund.org/en/>)

**Table S1.** Details about informants

| **Informant*** | **Number** | **Codename/pseudonym**** |
| --- | --- | --- |
| Global health expert | 1 | E1 |
| US Pharmacopeia expert | 1 | E2 |
| Ministry of Health (Ghana) – Procurement and Traditional Medicine Practice Council (TMPC) | 2 | E3, E4 |
| Pharmaceutical Society of Ghana representative | 1 | E5 |
| Pharmacy Council Ghana representative | 1 | E6 |
| Food and Drugs Authority (FDA) Ghana | 1 | E7 |
| Ghana Standards Authority (GSA) | 1 | E8 |
| University researcher (Pharmacy) | 2 | E9, E10 |
| Pharmacists | 2 | P1, P2 |
| Registered chemists/ herbalists (shops) | 4 | S1-S4 |
| Open Street Market vendors | 8 | V1-V8 |
| Consumers | 10 | C1-C10 |
| **Total number of informants** | **34** | **-** |

*E = expert; P = pharmacist; S = shop owner/registered chemist/herbalist; V = open street market vendor; C = consumer
